# Diterpene Synthases and Their Responsible Cyclic Natural Products

**DOI:** 10.1007/s13659-014-0012-8

**Published:** 2014-04-18

**Authors:** Hai-Yan Gong, Ying Zeng, Xiao-Ya Chen

**Affiliations:** 1 State Key Laboratory of Phytochemistry and Plant Resources in West China, Kunming Institute of Botany, Chinese Academy of Sciences, 132 Lanhei Road, Kunming, 650201 China; 2 National Key Laboratory of Plant Molecular Genetics, Institute of Plant Physiology and Ecology, Shanghai Institutes for Biological Sciences, Shanghai, 200032 China; 3 University of Chinese Academy of Sciences, Beijing, 100049 China

**Keywords:** Biosynthesis, cDNA, Cyclase, Diterpenoid, Eriocalyxin B

## Abstract

This review provides an overview of diterpene synthases which were initially identified via genetic and/or biochemical means, traversing all organisms researched to date.

## Introduction

Naturally occurring cyclic diterpenoids are the foundation for a number of biologically important compounds, which confer anti-microbial and anti-tumor action i.e. tanshinones, Taxol, and platensimycin. They are generally derived from the linear primary metabolite (*E,E,E*)-geranylgeranyl diphosphate (GGPP) by diterpene synthases (diTPS). These enzymes undergo complex electrophilic cyclizations and/or rearrangements leading to diverse backbone structures. According to the established general patterns of diterpene biosynthesis, diTPS can be divided into two mechanistically distinct categories: class I and class II enzymes. Structural and mechanistic studies have demonstrated that class I diTPS contain a characteristic aspartate (D)-rich DDXXD motif that binds divalent metal ions required for the catalysis of diphosphate ionization. On the other hand, class II diTPS exhibit a typical DXDD motif which is abundant in aspartate, that confers a role in initiating cyclization by protonating the terminal C=C double bond of GGPP [1, 2]. Usually, the class II diTPS yield terpene diphosphates that can serve as substrates of class I diTPS. Highly reactive carbocation intermediates arise from the cleavage of diphosphate group on the substrate by class I diTPS that end the cyclization cascade, by quenching the carbocation via deprotonation or electrophilic attack by water. A significant amount of cyclic diterpenoids are known to originate biosynthetically from dual cyclization and/or rearrangement reactions, which proceed via a bicyclic diphosphate intermediate [2]. In the past decades, there has been some remarkable progress in the research of biosynthetic genes and enzymes responsible for scaffold cyclization to diterpenoids in plants, fungi, bacteria, and even animals. This review attempts to describe diTPS cloned as cDNAs and functionally characterized in all organisms researched to date, and further focusing on those first identified genetically and/or biochemically. Information regarding chemical structures of enzyme products and their relevant diterpenoid natural products will be listed in tables according to hydrocarbon backbones of the natural diterpenoids, in parallel with organisms and enzyme accession numbers released on GenBank. The same enzyme relevant to a different diterpenoid product is specifically included here.

## Enzymes for Bicyclic Hydrocarbon Skeletons (Table 1)

The first diTPS of prokaryotic origin was reported in *Streptomyces griseolosporeus* MF730-N6 (currently referred to as *Kitasatospora griseola*), a diterpenoid anti-biotic terpentecin (**1**) producer. Using the general diterpenoid precursor GGPP as its substrate, the class II terpentedienyl diphosphate synthase produces a bicyclic terpentedienyl diphosphate (PP) which is then transformed to terpentetriene by the class I terpentetriene synthase. These two enzymes are involved in terpentecin (**1**) biosynthesis [3, 4]. In *Mycobacterium tuberculosis* H37, a bacterium causing fatal infections of tuberculosis, via the bicyclization of GGPP by the class II Rv3377c, produces halimadienyl (tuberculosinyl) PP that undergoes subsequent ionization-dependent re-arrangement catalyzed by the class I Rv3378c to give tuberculosinol {5(6),13-halimadiene-15-ol} and isotuberculosinol (**2**, also known as nosyberkol) [5–7]. Interestingly, the two diTPS genes are specifically found in the virulent species, but not in the avirulent species [7].

**Table 1:** Enzymes for bicyclic hydrocarbon skeletons

*Genus species* (organisms), enzymes/GenBank accession numbers	Enzyme products	Natural diterpenoids
*Kitasatospora griseola* (bacterium) [3, 4]; terpentedienyl diphosphate synthase/BAB39206; terpentetriene synthase/BAB39207		
*Mycobacterium tuberculosis* (bacterium) [5–7]; halimadienyl (tuberculosinyl) diphosphate synthase/CCP46198 (Rv3377c); tuberculosinol and isotuberculosinol synthase/CCP46199 (Rv3378c)		
*Selaginella moellendorffii* (lycophyte) [8]; labda-7,13*E*-dien-15-ol synthase (bifunctional)/AEK75338		
*Abies balsamea* (gymnosperm) [9]; *cis*-abienol synthase (bifunctional)/AEL99953		
*Nicotiana tabacum* (dicot) [10]; labda-13*E*-8-ol diphosphate synthase/CCD33018; *cis*-abienol synthase/CCD33019		
*Cistus creticus* subsp. *creticus* (dicot) [11]; labda-13*E*-8-ol diphosphate synthase/ADJ93862	labda-13*E*-8-ol PP	
*Salvia sclarea* (dicot) [12]; labda-13*E*-8-ol diphosphate synthase/AFU61897; sclareol and manool synthase/AFU61898	labda-13*E*-8-ol PP sclareol	

The basal vascular land plant lycophyte *Selaginella moellendorffii* contains a class I/II bi-functional enzyme labda-7,13*E*-dien-15-ol synthase that directly generates the endocyclic double bond and the hydroxyl group to form the bicyclic product labda-7,13*E*-dien-15-ol (**3**) [8]. The labdanoid diterpene alcohol *cis*-abienol (**4**) is a major component of the aromatic oleoresin of the gymnosperm balsam fir (*Abies balsamea*) and serves as a valuable bioproduct material for the fragrance industry. A bi-functional class I/II *cis*-abienol synthase (AbCAS) was discovered using high-throughput 454 transcriptome sequencing and metabolite profiling of balsam fir bark tissue [9]. AbCAS-catalyzed formation of *cis*-abienol (**4**) proceeds via cyclization and hydroxylation at carbon C-8 of a postulated carbocation intermediate in the class II active site, followed by cleavage of the diphosphate group and termination of the reaction sequence without further cyclization in the class I active site [9]. In the leaves of the angiosperm tobacco (*Nicotiana tabacum*) there are two consecutive reactions catalyzed by separate diTPS which lead to the production of *cis*-abienol (**4**). Accordingly, a labda-13*E*-8-ol PP synthase (NtCPS2) encodes a class II diterpene synthase that produces labda-13*E*-8-ol PP from GGPP; NtABS encodes a class I diterpene synthase that uses labda-13*E*-8-ol PP to form *cis*-abienol (**4**) [10].

Another labda-13*E*-8-ol PP synthase from *Cistus creticus* subsp. *creticus* was found to catalyze cyclization of GGPP to labda-13*E*-8-ol (copal-8-ol) PP, a likely intermediate in the biosynthesis of the oxygen-containing labdane-type diterpenes labdenediol (**5**) and manoyl oxide (**6**) abundant in the resin of this plant [11]. The bicyclic sclareol (**7**) extracted from cultivated clary sage (*Salvia sclarea*) is a diterpene natural product of high value for the fragrance industry and a valued starting material for semi-synthesis of numerous commercial substances, including production of Ambrox^®^. Similar to the labdanoid alcohols in two dicot plants mentioned above, sclareol (**7**) and manool (**8**) were produced in a two-step process mediated sequentially by labda-13*E*-8-ol PP synthase and sclareol/manool synthase [12]. Only a few diTPS generate the hydroxylated diterpenoids without requirement for additional oxidation by enzymes like cytochrome P450 monooxygenases.

## Enzymes for Tricyclic Hydrocarbon Skeletons (Table 2)

The *ent*-copalyl diphosphate (CPP) synthase and pimara-9(11),15-diene synthase from *Streptomyces* sp. strain KO-3988, is responsible for *ent*-CPP and pimara-9(11),15-diene formation, respectively, and were confirmed to participate in viguiepinol (**9**) {3-hydroxypimara-9(11),15-diene} biosynthesis by a heterologous expression experiment [13, 14]. Notably, Rv3378c from *Mycobacterium tuberculosis* H37 was found to encode an unusual halimadienyl PP specific class I diterpene synthase that is primarily involved in the production of the tricyclic edaxadiene (**10**) [15]. Platensimycin (PTM, **11**) and platencin (PTN, **12**) harbor diterpene moieties were isolated from *Streptomyces platensis* strains. They exhibit inhibitory activity toward bacterial and mammalian fatty acid synthases and have great potential in anti-bacterial and anti-diabetic drug development [16, 17]. Their biosyntheses are controlled by dedicated *ent*-kaurene synthase (PtmT3) and *ent*-atiserene synthases (PtmT1 and PtnT1) that channel the common *ent*-CPP precursor formed by the *ent*-CPP synthases (PtmT2 and PtnT2) to the characteristic PTM and PTN scaffolds, respectively. Therefore, the class I diterpene synthases PtmT1 and PtnT1 represent the first *ent*-atiserene synthases discovered, unveiling a previously undescribed pathway for the biosynthesis of diterpenoid natural products from the common precursor *ent*-CPP [16, 17]. The lysophospholipase inhibitor cyclooctatin (**13**) from *Streptomyces melanosporofaciens* MI614-43F2 is characterized by a 5-8-5 fused ring system. Sequence analysis in combination with sub-cloning and gene deletion revealed that the CotB2 gene encodes an enzyme that acts as a class I diTPS to produce cyclooctat-9-en-7-ol from GGPP [18]. The reaction is most likely to be initiated by ionization of the diphosphate of GGPP and followed by proton-induced cyclization, employing a similar mechanism underlying the taxadiene formation in taxol biosynthesis [18]. Harboring the same 5-8-5 fused ring scaffold, fusicoccins (**14**) are the major metabolites produced by the plant-pathogenic fungus *Phomopsis* (originally known as *Fusicoccum*) *amygdali* (Del.). They function as potent activators of plasma membrane H^+^-ATPase in plants and also exhibit unique biological activity in animal cells. The tricyclic hydrocarbon skeleton of fusicoccin A (**14**) is biosynthesized by an unusual multifunctional enzyme, *P. amygdali* fusicoccadiene synthase (PaFS) showing both prenyltransferase and terpene synthase activities [19]. Equipped with a terpene synthase domain at the N terminus and a GGPP synthase domain at the C terminus, PaFS is able to form GGPP and the resulting (+)-fusicocca-2,10(14)-diene using C_5_ isoprene units as precursors [19]. Another fusicoccadiene synthase was identified in the fungus *Alternaria brassicicola* strain ATCC 96836 for the brassicicene C (**15**) biosynthesis in a similar manner to that of PaFS [20].

**Table 2:** Enzymes for tricyclic hydrocarbon skeletons. (Color table online)

*Genus species* (organisms), enzymes/GenBank accession numbers	Enzyme products	Natural diterpenoids
*Streptomyces* sp. (bacterium) [13, 14]; *ent*-CPP synthase/BAD86797; pimara-9(11),15-diene synthase/BAD86798		
*Mycobacterium tuberculosis* (bacterium) [15]; halimadienyl diphosphate synthase/CCP46198 (Rv3377c); edaxadiene synthase/CCP46199 (Rv3378c)		
*Streptomyces platensis* (bacterium) [16, 17]; *ent*-CPP synthases/ACO31276 (PtmT2), ADD83015 (PtnT2); *ent*-kaurene synthase/ACO31279 (PtmT3); *ent*-atiserene synthase/ACO31274 (PtmT1), ADD83014 (PtnT1); Diterpene moieties are highlighted in red		
*Streptomyces melanosporofaciens* (bacterium) [18]; cyclooctat-9-en-7-ol synthase/BAI44338 (CotB2)		
*Phomopsis amygdali* (fungus) [19]; fusicoccadiene synthase/BAF45924		
*Alternaria brassicicola* (fungus) [20]; fusicoccadiene synthase/BAI44849		
*Abies grandis* (gymnosperm) [21]; abietadiene synthase/AAB05407		
*Picea abies* (gymnosperm) [22, 23]; isopimara-7,15-diene synthase/AAS47690; levopimaradiene/abietadiene synthase/AAS47691		diterpene resin acids (rosin)
*Ginkgo biloba* (gymnosperm) [24]; levopimaradiene synthase/AAL09965		
*Taxus brevifolia* (gymnosperm) [25, 26]; taxadiene synthase/AAC49310		
*Salvia miltiorrhiza* Bunge (dicot) [27]; CPP synthase/ABV57835; miltiradiene synthase/ABV08817		
*Oryza sativa* (monocot) [28–30]; *ent*-CPP synthase/AAT11021 (OsCPS2), BAD42452 (OsCyc2); *syn*-CPP synthase/BAD42451 (OsCyc1), AAS98158 (OsCPS4)		phytoalexins
*Oryza sativa* (monocot) [31, 32] *ent*-cassa-12,15-diene synthase/BAC56714 (OsDTC1), ABH10734 (OsKSL7); A: R1=R2=H, R3=OH, R4=R5=O B: R1=OH, R2=H, R3=OH, R4=H, R5=OH C: R1=OH, R2=R3=R4=H, R5=OH D: R1=H, R2=R3=O, R4=R5=H E: R1=OH, R2=R3=H, R4=R5=O	(*ent*-CPP)	
*Oryza sativa* (monocot) [32, 33]; *ent*-sandaracopimaradiene synthase/ABH10735 (OsKSL10), BAD54752 (OsKS10) A: R1=H, R2=OH, R3=CH3, R4=R5=O, R6=H B: R1=R2=O, R3=CH3, R4=OH, R5=R6=H C: R1=R2=O, R3=CH3, R4=R5=O, R6=H D: R1=H, R2=OH, R3=CH3, R4=R5=H, R6=OH E: R1=H, R2=OH, R3=CH3, R4=R5=H, R6=OH F: R1=H, R2=OH, R3=CH2OH, R4=R5=R6=H	(*ent*-CPP)	
*Oryza sativa* (monocot) [32, 34, 35]; *ent*-pimara-8(14),15-diene synthase/BAE72100 (OsKS5, OsKSL5j)	(*ent*-CPP)	currently unknown
*Oryza sativa* (monocot) [33, 36]; *syn*-pimara-7,15-diene synthase/BAD54751(OsKS4), AAU05906 (OsKSL4)	(*syn*-CPP)	
*Pseudopterogorgia elisabethae* (gorgonian, marine invertebrate) [37, 38]; natively purified enzyme		

Diterpene resin acids (**16**, rosin) secreted by the gymnosperm conifer species are important defense compounds against herbivores and pathogens. The abietadiene synthase from grand fir (*Abies grandis*; AgAS) is the first diterpene synthase in conifers to be cloned and functionally characterized, and also a bifunctional diTPS catalyzing both protonation-initiated and ionization-initiated cyclization steps for rosin biosynthesis [21]. The AgAS’s homologues from Norway spruce (*Picea abies*), isopimara-7,15-diene synthase and levopimaradiene/abietadiene synthase (PaLAS), produce isopimara-7,15-diene and a mixture of levopimaradiene, abietadiene, neoabietadiene, and palustradiene, respectively, when incubating with GGPP as substrate [22]. The initial product of PaLAS, however, may be epimers of the thermally unstable allylic tertiary alcohol 13-hydroxy-8(14)-abietene that is likely by dehydration to yield a mixture of the above four diterpene hydrocarbons [23]. Another homologue from *Ginkgo biloba* encodes a bifunctional levopimaradiene synthase catalyzing the initial cyclization step in ginkgolide (**17**) pathway [24]. Biosynthesis of the anticancer drug Taxol (**18**) in *Taxus* (yew) species involves 19 steps from GGPP that first cyclizes to the taxane skeleton by taxadiene synthase [25, 26].

Tanshinones (**19**) are abietane-type norditerpenoid quinone natural products in the Chinese medicinal herb Dānshēn (*Salvia miltiorrhiza* Bunge). The CPP synthase and miltiradiene synthase cDNAs have been cloned from the induced root tissue and demonstrated to be involved in the biosynthesis of tanshinones [27]. In the monocot plant rice (*Oryza sativa*, *O. glaberrima*), the diterpenoid phytoalexins have been identified to derive via the common biosynthetic origins in the initiating dual cyclization and/or rearrangement of GGPP precursor. The *ent*-CPP synthase (OsCPS2/OsCyc2) paired with *ent*-cassa-12,15-diene and *ent*-sandaracopimaradiene synthases, respectively, give rise to phytocassanes A–E (**20**) and oryzalexins A–F (**21**) [28–33]. An *ent*-CPP specific *ent*-pimara-8(14),15-diene synthase was discovered in spite of its currently unknown products [32, 34, 35]. In addition, the dual phytoalexin/allelochemical momilactones (**22a** and **22b**) are formed by tandem catalysis of *syn*-CPP synthase (OsCPS4/OsCyc1) and *syn*-pimara-7,15-diene synthase [33, 36]. The only animal diTPS which has been reported is a natively purified enzyme from a marine invertebrate gorgonian (*Pseudopterogorgia elisabethae*), which shows elisabethatriene synthase activity presumably linked to the formation of pseudopterosins (**23**) [37, 38].

## Enzymes for Tetracyclic Hydrocarbon Skeletons (Table 3)

The ubiquitous gibberellin phytohormones (**24a**-**c**) are tetracyclic diterpenoids produced via distinct biosynthetic pathways by plants, fungi, and bacteria. Like in plants, the bacterium *Bradyrhizobium japonicum* was demonstrated to encodes for separate *ent*-CPP and *ent*-kaurene synthases for gibberellic acid GA_3_ production (**24a**) [39]; while fungi use only a single bi-functional *ent*-kaurene synthase for GA_1_ (**24b**) [40]. Aphidicolin (**25**) isolated from the fungus *Phoma betae* PS-13 has a unique hydrocarbon skeleton and remarkable bio-activity such as anti-tumor and specific inhibition of DNA polymerase α. Aphidicolan-16β-ol synthase was identified as a bifunctional diTPS involved in the biosynthesis of aphidicolin (**25**) from GGPP via *syn*-CPP [41]. However, the metabolite phyllocladan-11α,16α,18-triol (**26**) is likely to result from a two-step cyclization via CPP synthase coupled with phyllocladan-16α-ol synthase in the fusicoccin (**14**)-producing fungus *Phomopsis amygdali* [19, 42]. Using homology based PCR of GGPP synthase genes and the subsequent genome walking in the fungus *Phomopsis amygdali* N2, phomopsene synthase was identified and led to discovery of a new diterpene, methyl phomopsenonate (**27**) [43].

**Table 3:** Enzymes for tetracyclic hydrocarbon skeletons

*Genus species* (organisms), enzymes/GenBank accession numbers	Enzyme products	Natural diterpenoids	
*Bradyrhizobium japonicum* (bacterium) [39]; CPP synthase/BAC47414; kaurene synthase/BAC47415			
*Phaeosphaeria sp.* (fungus) [40]; *ent*-kaurene synthase/BAA22426			
*Phoma betae* PS-13 (fungus) [41]; aphidicolan-16β-ol synthase/BAB62102			
*Phomopsis amygdali* (fungus) [42]; CPP synthase/BAG30962; phyllocladan-16α-ol synthase/BAG30961			
*Phomopsis amygdali* (fungus) [43]; phomopsene synthase/AB252833 (DDBJ: mRNA)			
*Physcomitrella patens* (bryophyte) [44–46]; *ent*-CPS/KS/BAF61135	*ent*-kaurene; 16α-hydroxy-*ent*-kaurane		
*Arabidopsis thaliana* (dicot) [47]; *ent*-CPP synthase/AAA53632 *Cucurbita maxima* (dicot) [48]; *ent*-kaurene synthase/AAB39482			
*Stevia rebaudiana* (dicot) [49]; *ent*-CPP synthase/AAB87091; *ent*-kaurene synthase/AAD34294	*ent*-CPP *ent*-kaurene		
*Isodoneriocalyx* (dicot) [50]; *ent*-CPP synthase/AEP03175	*ent*-CPP	*Isodonent*-kauranoids	
*Zea mays* (monocot) [51, 52]; *ent*-CPP synthase (AN2)/AAT70083, AAT70084	*ent*-CPP		
*Oryza sativa* (monocot) [28–30] *ent*-CPP synthase/AAT11021 (OsCPS2), BAD42452 (OsCyc2) *syn*-CPP synthase/BAD42451 (OsCyc1), AAS98158 (OsCPS4)		phytoalexins	
*Oryza sativa* (monocot) [32, 34, 35] *ent*-isokaurene synthase: OsKS6/BAE72101, OsKSL6/ABH10733, OsKSL5i/ABH10732	(*ent*-CPP)		
*Oryza sativa* (monocot) [53]; *syn*-stemar-13-ene synthase/BAD34478 (OsKSL8)	(*syn*-CPP)		
*Oryza sativa* (monocot) [54]; *syn*-stemodene synthase/AAZ76733 (OsKSL11)	(*syn*-CPP)	currently unknown	
*Sapium sebiferum* (dicot) [55]; casbene synthase/ADB90272			
*Euphorbia esula* (dicot) [55]; casbene synthase/ADB90273	Casbene		

In the basal vascular land plant moss *Physcomitrella patens*, a bifunctional *ent*-CPS/KS directly generates the *ent*-kaurene skeleton from GGPP to give *ent*-kaurene (**28**), 16α-hydroxy-*ent*-kaurane (**29**) and *ent*-kaurenoic acid (**30**) [44–46]. Instead, most angiosperms use normal, *ent*, or *syn*-CPP synthase and kaurene synthase or kaurene synthase-like (KSL) for the two sequential cyclization reactions that initiate biosyntheses of gibberellins such as GA_4_ (**24c**) and labdane-related diterpenoids. The plant gibberellin diTPS were the first identified in *Arabidopsis thaliana* and *Cucurbita maxima*, respectively [47, 48]. Interestingly, the intensely sweet steviol (**31**) producing plant *Stevia rebaudiana* was found to share the same *ent*-CPP synthase and *ent*-kaurene synthase for both gibberellin and steviol (**31**) biosyntheses [49]. In contrast, *Isodon* plants are most likely to recruit novel a diTPS that functions as an *ent*-CPP synthase specific for the production of *ent*-kaurane diterpenoids (also *ent*-kauranoids) like eriocalyxin B (**32**) [50]. The induced accumulation of six *ent*-kaurane–related diterpenoid phytoalexins, collectively termed kauralexins (**33**) was observed in maize in response to stem attack by the European corn borer (*Ostrinia nubilalis*) and fungi. The maize *An2* encoding an *ent*-CPP synthase, is probably relevant to kauralexin (**33**) production [51, 52]. Besides tri-cyclic diterpenoid phytoalexins as described in Table 2, rice produces tetracyclic phytoalexins such as oryzalides (**34**) and oryzalexin S (**35**). The *ent*-CPP synthase (OsCPS2/OsCyc2) and *ent*-isokaurene synthase in tandem catalyze the formation of hydrocarbon skeletons of oryzalides (**34**) [32; 34, 35]; wherase the *syn*-CPP synthase (OsCPS4/OsCyc1) coupled with *syn*-stemar-13-ene synthase generate those of oryzalexin S (**35**) [53]. Despite the demonstration of *syn*-CPP specific *syn*-stemodene synthase activity in rice, its relevant natural product has remained elusive [54]. The latent HIV-1 activator prostratin (and related 12-deoxyphorbol esters), the analgesic resiniferatoxin, and the anti-cancer drug candidate ingenol 3-angelate are Euphorbiaceae diterpenoids of medical interest and likely derived from casbene. Casbene synthase genes were considered for carbon skeletons formation of 12-deoxyphorbol 13-benzoate (**36**) and ingenol (**37**), respectively in *Sapium sebiferum* and *Euphorbia esula* [55].

## Enzymes for Macrocyclic Hydrocarbon Skeletons (Table 4)

Two novel class I diTPS DtcycA and DtcycB, derived from *Streptomyces* sp. SANK 60404, are capable of forming multiple diterpene products with macrocyclic skeletons. The reaction products catalyzed by DtcycA were determined to be the isopropylidene isomer of cembrene C (**38**) and (*R*)-nephthenol (**39**); while the outcome of DtcycB included (*R*)-nephthenol (**39**), (*R*)-cembrene A (**40**), and a novel diterpene {(4*E*,8*E*,12*E*)-2,2,5,9,13-pentamethylcyclopentadeca-4,8,12-trien-1-ol} (**41**) [56]. However, none of these diterpene products were detected in the culture broths under any of the culture conditions used in this study, as revealed by GC–MS analysis. Furthermore, DtcycA and DtcycB show no sequence similarity to cembratrieneol synthase for cembratrienediol (**42**) in *Nicotiana tabacum* [57] or to neocembrene (**43**) (also known as cembrene A) synthase (CAS2) from castor bean (*Ricinus communis*) [55]. Casbene (**44**) is a macrocyclic diterpene hydrocarbon that serves as a phytoalexin in castor bean [58]. The casbene synthase from castor bean is the first diTPS cloned as cDNA as early as in 1994 [59].

**Table 4:** Enzymes for macrocyclic hydrocarbon skeletons

*Genus species* (organisms), enzymes/GenBank accession numbers	Enzyme products	Natural diterpenoids
*Streptomyces* sp. (bacterium) [56]; DtcycA/BAM78697; DtcycB/BAM78698		currently unknown
*Nicotiana tabacum* (dicot) [57]; cembratrieneol synthase/AAL25826		
*Ricinus communis* (dicot) [55]; neocembrene synthase (CAS2)/XP_002513334	Neocembrene	
*Ricinus communis* (dicot) [55, 59]; casbene synthase/EEF48743 (CAS1), EEF48746 (CAS3)	Casbene	

In summary, angiosperm plants and bacteria generally share the common two-step process that shapes the hydrocarbon cyclic skeletons of diterpenoids by two mono-functional diTPS in tandem catalysis. Such a correlation seems plausible considering that the plastidial MEP (2-*C*-methyl-D-erythritol phosphate) pathway dominates the C_5_ isoprene precursor supplies and plastids are thought to have originated from endosymbiotic cyanobacteria. Conversely, bi-functional class I/II diTPS are more likely found in gymnosperms, basal vascular land plants, and fungi. Currently, only class I diTPS can produce macrocyclic skeletons of natural diterpenoids. In the near future, ‘Omics’ approaches and metabolite profiling can be extensively exploited in an effort to reveal genes and enzymes responsible for cyclization and subsequent modification reactions that take place in diterpenoid biosyntheses of plants, fungi, and bacteria.
